# Crown Ether Grafted Graphene Oxide/Chitosan/Polyvinyl Alcohol Nanofiber Membrane for Highly Selective Adsorption and Separation of Lithium Ion

**DOI:** 10.3390/nano11102668

**Published:** 2021-10-11

**Authors:** Xudong Zheng, Ang Li, Jie Hua, Yuzhe Zhang, Zhongyu Li

**Affiliations:** 1 School of Environmental and Safety Engineering, Changzhou University, Changzhou 213164, China; la122240180@gmail.com (A.L.); msi893803760@gmail.com (J.H.); yuzhez@cczu.edu.cn (Y.Z.); 2 Jiangsu Engineering Research Center of Petrochemical Safety and Environmental Protection, Changzhou 213164, China

**Keywords:** crown ether grafting, selective adsorption, composite membrane

## Abstract

Nanofiber membranes were successfully prepared with crown ether (CE) functionalized graphene oxide (GO), chitosan (CS), and polyvinyl alcohol (PVA) by low-temperature thermally induced liquid–liquid phase separation. The physical and chemical properties and adsorption performance of nanofiber membrane were studied through SEM, FT-IR, XRD, and static adsorption experiments. The results show that the specific surface area of the nanofiber membrane is as high as 101.5 m^2^∙g^−1^. The results of static adsorption experiments show that the maximum adsorption capacity of the nanofiber membrane can reach 168.50 mg∙g^−1^ when the pH is 7.0. In the selective adsorption experiment, the nanofiber membrane showed high selectivity for Li^+^ in salt lake brine. After five cycles, the material still retains 88.31% of the adsorption capacity. Therefore, it is proved that the material has good regeneration ability.

## 1. Introduction

In recent years, with people’s increasing attention to environmental pollution and the continuous advancement of science and technology [1], electrification has gradually become a major trend in the development of the global automotive industry, coupled with the popularization of consumer electronics such as mobile phones, tablets, and computers [2]. Lithium, as the core material of electric vehicle power batteries and the key material of lithium-ion batteries for consumer electronics products, has broad development prospects [3]. The lithium material industry spans the three strategic emerging industries of new materials, new energy vehicles, and new energy in China [4]. The development of the new energy automobile industry requires speeding up the research and development, promotion, and application of core technologies of key components and materials such as high-performance power batteries and motors, forming an industrialized system. The demand for lithium batteries will rise rapidly [5], and the market demand for lithium will rise sharply. The development and utilization of large lithium resources has become an important issue to ensure sufficient lithium resources [6]. Salt lake brine is the main source of lithium extraction technology at present, and the common feature of salt lake lithium resources in China is the high ratio of magnesium to lithium [7]. Therefore, the development of lithium extraction and separation technology suitable for salt lake brines with high magnesium-to-lithium ratio in China has very important economic value and strategic significance [8].

At present, the common methods of extracting lithium are mainly the precipitation method, solvent extraction method, electrodialysis method, and adsorption method [9]. Among them, the precipitation method is more suitable for the extraction of lithium from salt lake brines where the magnesium–lithium ratio is relatively low [10]. For the case where the magnesium–lithium ratio is too high, the precipitant consumption is too large and the economy is poor. The solvent extraction method represents the most in-depth study of the neutral phosphorus-containing TBP extraction system, but it has not been industrialized because of the serious corrosion of TBP to the equipment and the easy dissolution during the stripping process [3]. The emerging electrodialysis technology also needs to be improved owing to research work such as membrane life and operating costs under long-term operating conditions [11]. In contrast, the adsorption method is widely used because of its simple operation and high recovery rate. The adsorption material is the core of the adsorption and extraction of lithium [12]. Many studies have found that ion sieve adsorbents have high lithium adsorption capacity and selectivity, but they are usually powdery, with poor fluidity and permeability, and a low dissolution rate during acid elution regeneration, making them difficult to use for industrial applications. Therefore, we are committed to finding a low-cost, selective, and stable lithium adsorbent [13].

The crown ether as the host has a certain dipole moment and can interact with the guest ion to coordinate, owing to the existence of electron-donating oxygen atoms. The hole diameter of the crown ether ligand is 1.223–1.534 Å, and the diameter of Li^+^ is 1.22 Å, which matches the hole size, and can completely enter the hole and form a strong electrostatic interaction force with the crown ether oxygen [14]. However, crown ethers usually exist in the form of small molecules in liquid form, and they are usually introduced as ligands into functional groups, so that they are easily connected to the matrix through chemical bonds to form a composite adsorbent with a stable structure [15].

Graphene oxide (GO) is a new type of carbon nanomaterial, which not only has high mechanical strength and electron mobility, but also has a large specific surface area and abundant oxygen-containing functional groups [16]. It not only provides effective adsorption sites, but also can be used as a solid carrier for easy introduction of small molecule ligands. Chitosan (CS) macromolecules are rich in functional groups such as hydroxyl and amino groups [17], have strong coordination ability with heavy metal ions, and have good adsorption performance for heavy metals. Chitosan is cheap and easy to obtain, and it is a green and pollution-free material [18]. Polyvinyl alcohol (PVA) is used in many biological materials because of its low cost, low toxicity, high mechanical strength, excellent biocompatibility, and chemical stability [19]. Thanks to the good biocompatibility of chitosan and polyvinyl alcohol, some studies have prepared it into a hydrogel, but this structure has a small specific surface area and can easily to self-swell and shrink, thereby reducing the active sites [20]. The preparation of nanofiber membranes by electrospinning technology and the low-temperature thermally induced phase separation method seems to be a good solution. However, electrospinning technology is usually combined with high concentrations of toxic solvents.

Low temperature thermal induced liquid–liquid phase separation is a method used to prepare porous nanofiber membranes [21]. The polymer is dissolved in the mixed solvent and the temperature and time conditions are controlled. At the temperature below the freezing point of the mixed solvent, the polymer will form two liquid phases. The polymer is a continuous phase and the solvent is a dispersed phase after phase separation [22]. Therefore, it is very important to control the freezing point of mixed solvents [23]. It is well known that adding ethylene glycol to the solvent can lower the freezing point of the solvent and can initiate two-phase separation during the freeze-drying process to form a nanofiber porous structure [24].

In this paper, a step-by-step method is used to first graft the crown ether onto the surface of graphene oxide to obtain functioned graphene oxide (GO-CE). Then, GO-CE, CS, and PVA were combined together, ethylene glycol was used to lower the freezing point of the solvent, and a composite membrane with nanofiber structure was prepared by low-temperature induced phase separation technology, which achieved high-efficiency selectivity to Li^+^ adsorption separation. The composite film was characterized by scanning electron microscope (SEM), Fourier transform infrared spectroscopy (FTIR), N_2_ adsorption-desorption (BET), and thermogravimetric analyzer (TG). Moreover, through a series of static and dynamic adsorption experiments and simulations of actual samples, the adsorption and selection properties of Li^+^ on the material are studied.

## 2. Experimental Section

### 2.1. Materials

The reagents used in this experiment are as follows: graphene oxide and polyvinyl alcohol. Chitosan was purchased from Thane Chemical Technology Co., Ltd. (Shandong, China), with a degree of deacetylation >95% and a viscosity of 100–200 mpa.s. 2-(Hydroxymethyl)-12-crown 4-ether was purchased from TCI Chemical Industry Development Co., Ltd. (Shanghai, China). Epichlorohydrin was purchased from Shanghai Lingfeng Chemical Reagent Co., Ltd. (Shanghai, China). Sodium hydride (60% dispersed in mineral oil), potassium bromide, and lithium chloride (anhydrous grade, 98%) were supplied by Aladdin Reagent Co., Ltd. (Shanghai, China). Acetic acid was provided by Shanghai Shenbo Chemical Co., Ltd. (Shanghai, China). Ethylene glycol, hydrochloric acid, and sodium hydroxide were obtained from Sinochem Reagent Co., Ltd. (Beijing, China). In addition, all reagents are analytically pure and can be used without further purification, and all experimental water is distilled water.

### 2.2. Sample Preparation

#### 2.2.1. Preparation of Graphene Oxide (GO−ECH)

**Figure 1:**
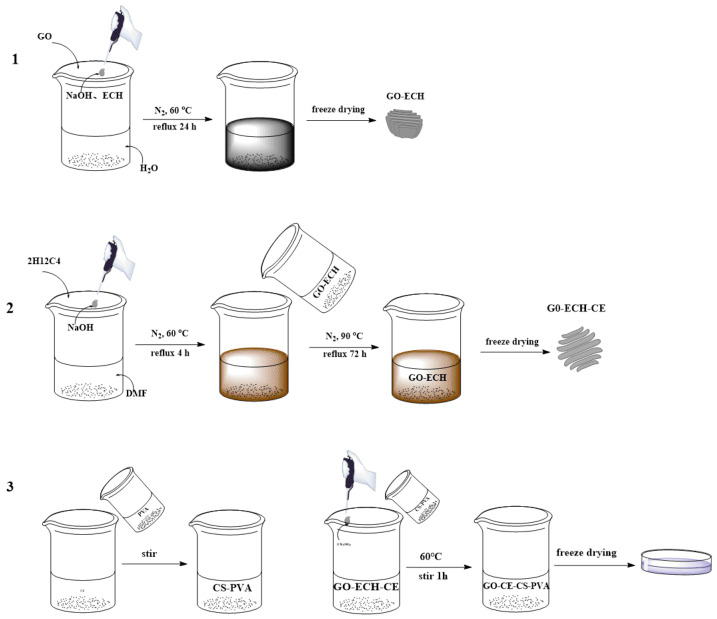
Experimental flow chart.

Here, 0.07 g of graphene oxide (GO) was dispersed in 70 mL of distilled water and 0.5 g of sodium hydroxide and 0.5 mL of epichlorohydrin were added, under N_2_ atmosphere, the temperature was controlled at 60 °C, and the reflux reaction was performed 24 h. Finally, a black mixture was obtained, and the black mixture was washed with distilled water to neutrality, and finally vacuum dried to obtain GO−ECH. As shown in the first step in Figure 1.

#### 2.2.2. Preparation of 2-(Hydroxymethyl)-12-Crown 4-Ether Graft Modified Graphene Oxide (GO−ECH−CE)

Here, 0.5 mL of 2-(hydroxymethyl)-12-crown 4-ether (2H12C4) was dissolved in 10 mL of N,N-dimethylformamide (DMF) at room temperature, and then 0.25 g of sodium hydride was added, in an N_2_ atmosphere The temperature was controlled at 60 °C, and the reflux reaction was carried out for 4 h to obtain a light brown mixed solution. Then, 0.05 g of the GO−ECH prepared in step (1) was dispersed in DMF and added to the above light brown mixed solution, continuing to control the temperature at 90 °C under N_2_ atmosphere, and reflux for 72 h. Then, it was rinsed with DMF until the supernatant was colorless and dry in vacuum to obtain GO−ECH−CE. As shown in the second step in Figure 1.

#### 2.2.3. Preparation of Crown Ether Grafted Graphene Oxide/Chitosan/Polyvinyl Alcohol Nanofiber Composite Film (GO–CE–CS–PVA)

Here, 1.0 g of polyvinyl alcohol (PVA) was dissolved in 20 mL of distilled water at 90 °C, and then 0.4 g of chitosan (CS) was dissolved in 20 mL of 2% (*v*/*v*) acetic acid solution at room temperature. The prepared PVA aqueous solution and chitosan solution were stirred at 60 °C for 1 h, so that the bubbles in the solution disappeared, and a uniformly mixed CS–PVA solution was obtained. Then, 0.2 g of the GO–CE prepared in step (2) was dispersed in 20 mL of distilled water, and the above-mentioned CS–PVA mixed solution was gradually added to the GO–CE dispersion, adding ethylene glycol (ethylene glycol/distilled water = 3/7) and stirring at 60 °C for 1 h, then cooling. Ultrasound was used to eliminate air bubbles in the solution. The solution was poured into a polytetrafluoroethylene mold and allowed stand at 253 K for 8 h to form a soft gel. Subsequently, the coagulant (1% NaOH solution) precooled at 273 K and was slowly added, and it was allowed to stand at 273 K for 12 h to grow and form a thin film. Then, the film was washed with distilled water, and the distilled water was replaced every 8 h until the supernatant became neutral. After freezing at 253 K for 2 h, it was freeze-dried at 223 K for 24 h to obtain GO-CE-CS-PVA. As shown in the third step in Figure 1.

#### 2.2.4. Preparation of Graphene Oxide/Chitosan/Polyvinyl Alcohol Nanofiber Composite Membrane (GO–CS–PVA) Membrane

The process was the same as that of crown ether grafted graphene oxide/chitosan/polyvinyl alcohol nanofiber composite film (GO–CE–CS–PVA), but with no grafting modification CE, GO.

#### 2.2.5. Repeat the Elution Procedure

The Li^+^ in the adsorbed composite film was eluted with 0.5 M hydrochloric acid and distilled water for two weeks. After elution, the composite membrane was vacuum-dried for the next adsorption cycle.

### 2.3. Adsorption Experiment

We performed pH, kinetics, isotherm, thermodynamics, selective adsorption, and repeatability experiments on GO–CE–CS–PVA and its control material GO−CS−PVA. All experimental results were measured by inductively coupled plasma optical emission spectrometer (ICP) to measure the concentration of Li^+^.

### 2.4. Characterization Instruments

A series of characterizations were carried out on the successfully prepared GO–CE–CS–PVA material. The morphology and microstructure of GO–CE–CS–PVA material were observed by scanning electron microscope (SEM, Xiemei Electronics Co., Ltd., Dongwan, Japan). The infrared spectrum (400–4000 cm^−1^) was recorded on a Fourier transform infrared spectrometer (FTIR, Xiou Technology Co., Ltd., Shanghai, China) to observe the functional groups in the GO–CE–CS–PVA material molecule. X-ray photoelectron spectroscopy (XPS, Kratos, Manchester, UK) was used to characterize the chemical elements of the GO–CE–CS–PVA material. The specific surface area of GO–CE–CS–PVA material under the N_2_ adsorption-desorption method was tested with a physical adsorption instrument. The thermal stability of GO–CE–CS–PVA material is measured by thermogravimetric analyzer (TG, TA Instruments Corporation USA, New Castle, DE, USA). Using the above-mentioned instruments, it is verified that GO–CE–CS–PVA material has good structural characteristics and ensures that the material has excellent adsorption performance for Li^+^.

## 3. Results and Discussion

### 3.1. Characterization Results

#### 3.1.1. Scanning Electron Microscope (SEM)

**Figure 2:**
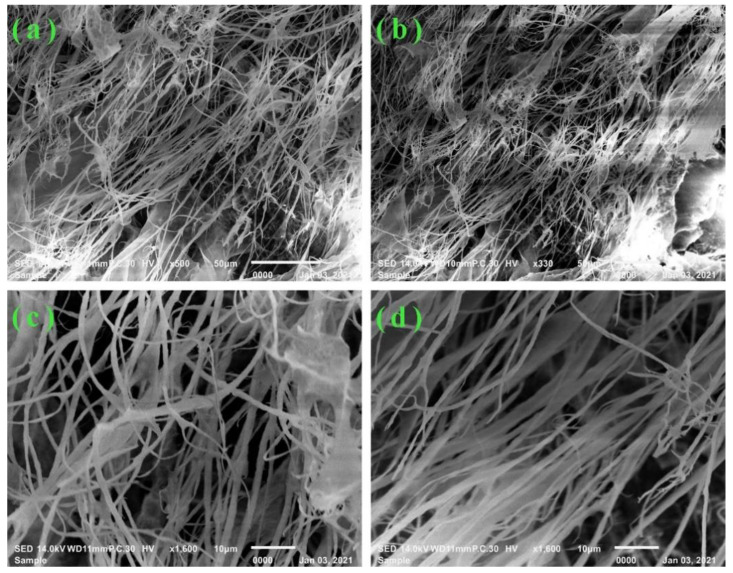
SEM images of polymer composite films at different magnification. (**a**–**d**) is the morphology observed at different magnification.

The surface morphology and structural characteristics of GO–CE–CS–PVA were studied by SEM observation. As shown in Figure 2, it can be seen that the inside of the composite membrane material is composed of nanofibers of different lengths, with an average diameter between 60 and 100 nm. From Figures (a) and (b), it can be observed that the obvious GO film is doped or covered on the nanofibers. In addition, we amplified (a) and (b) and obtained (c) and (d), which can more clearly and accurately observe that GO films are doped or covered on nanofibers. The formation of these heterogeneous nanofiber structures is attributed to the fact that glycol in the mixed solution can effectively reduce the freezing point of the solution, and the CS–PVA polymer was induced to undergo liquid–liquid phase separation instead of liquid–solid phase separation. In other words, at a low temperature, the thermodynamically unstable CS–PVA homogeneous polymer solution will be separated into polymer-rich phase and polymer-poor phase. In the polymer-rich phase, the polymer chains are folded and arranged to form a unique nanofiber structure. The formation of nanofiber structure and the doping of GO film increase the specific surface area of the composite membrane, increase the active adsorption sites on the adsorbent surface, and improve the mass transfer efficiency, which is helpful to improve the adsorption capacity of the composite membrane.

#### 3.1.2. Fourier Infrared Spectroscopy (FTIR)

**Figure 3:**
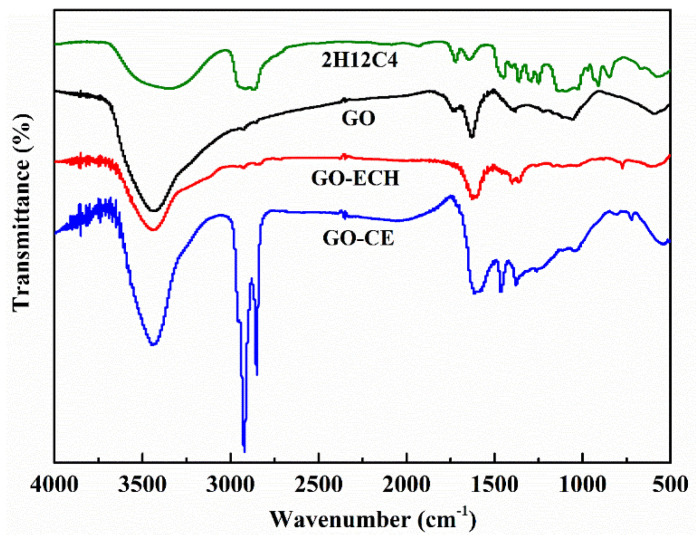
The FTIR of GO-CE, GO-ECH, GO, and 2H12C4.

In order to observe the changes of surface chemical groups of GO-CE during the grafting modification, the FTIR spectra of GO-CE and its control materials in the range of 400–4000 cm^−1^ were characterized. As shown in Figure 3, it can be seen that the infrared spectrum of each material has a wide and strong absorption peak at 3430 cm^−1^, which is the characteristic absorption peak of -OH, confirming that both GO and CE surfaces contain a large amount of -OH. The characteristic absorption peaks of pure GO at 1741 cm^−1^, 1630 cm^−1^, and 1060 cm^−1^ are attributed to the stretching vibration of C = O, C = C, and C-O, respectively. By epichlorohydrin, we first epoxidize the GO; compared with GO, GO-ECH new characteristic peaks appear at 771 cm^−1^. This peak is the characteristic absorption peak of epoxy group, explaining that the GO surface successfully connected to the epoxy ring. In addition, the characteristic peak of GO at 1741 cm^−1^ almost disappeared, indicating that the reaction took place on the carboxyl group. In the 2H12C4 spectrum, the strong absorption peak of 2800–3000 cm^−1^ comes from the stretching vibration of C-H, which is consistent with the result of GO–CE. In addition, 1460 cm^−1^ and 1370 cm^−1^ on GO–CE are the bending vibration peaks of C-H, and 1110 cm^−1^ is the characteristic absorption peak of C-O-C on crown ether ring. In conclusion, the FTIR curves analysis showed that 2H12C4 was successfully grafted on the GO surface through epichlorohydrin [25].

#### 3.1.3. N_2_ Adsorption-Desorption (BET)

**Figure 4:**
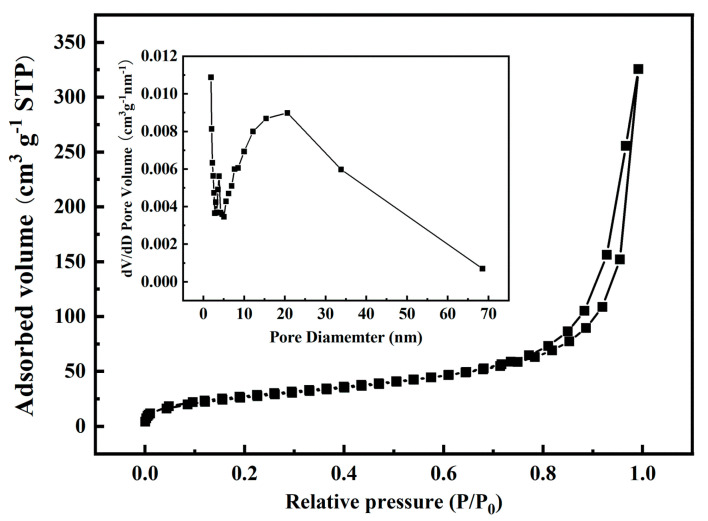
N_2_ adsorption-desorption isotherm diagram of GO–CE–CS–PVA.

The adsorption mechanism and specific surface area of the material were studied by the N_2_ adsorption-desorption analysis method. As shown in Figure 4, the adsorption isotherm of GO-CE-CS-PVA belongs to the IV type adsorption isotherm of the six adsorption isotherms classified by IUPAC. At a low relative pressure (0.0–0.05 P/P_0_), owing to the strong interaction on the surface of the adsorbent, the adsorption capacity rises rapidly, and the curve is upwardly convex, forming a single-layer adsorption [26]. After the adsorbent reaches the saturated adsorption capacity, multi-layer adsorption is gradually formed, and the relative pressure of the multi-layer adsorption is between 0.05 and 0.75 P/P_0_. As the pressure of the gas continues to increase, the pores of the gas agglomerates become larger and larger. The adsorption branch measured when the equilibrium pressure increases and the desorption branch measured when the pressure decreases, at certain relative pressure ranges, do not overlap and separate to form a ring. Under the same relative pressure, the adsorption capacity of the desorption branch is greater than the adsorption capacity of the adsorption branch, so the desorption isotherm will form a hysteresis loop above the adsorption isotherm. It can be seen from Figure 4 that the hysteresis loop belongs to the H_3_ type. When the relative pressure is close to the saturated vapor pressure, the adsorbate does not show adsorption saturation and cannot reach equilibrium. The pores of the material include flat slit structure, crack, and wedge structure. In addition, the BET specific surface area of the material is 101.5 m^2^·g^−1^, and the pore size of GO–CE–CS–PVA is concentrated in 0–10 nm, which belongs to micropore or mesoporous. This is attributed to the nanofiber structure constructed by the liquid–liquid phase separation technology realized by freeze-drying, and the larger BET specific surface area is beneficial to increase the adsorption capacity.

#### 3.1.4. Thermogravimetric Analysis (TG/DTG)

**Figure 5:**
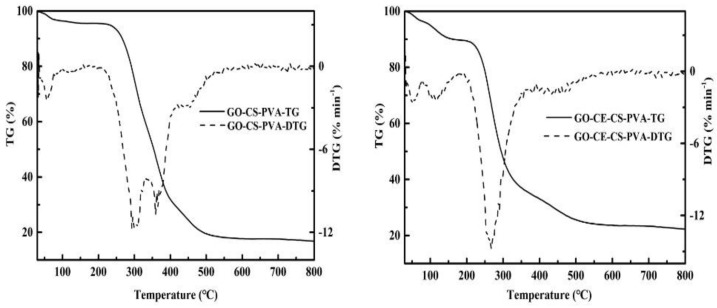
Thermogravimetric analysis diagram of GO–CE–CS–PVA and GO–CS–PVA.

In order to study the thermal stability of the material, we conducted a TG test under N_2_ atmosphere to analyze the relationship between the quality of the material and the temperature change in the temperature range of 30–800 °C. The thermogravimetric results of GO–CS–PVA and GO–CE–CS–PVA are shown in Figure 5. The weight loss at 100 °C in the figure is the loss of crystal water in the film. The weight loss of the material starts at about 250 °C and ends at about 500 °C. The weight lost at this stage is mainly chitosan and polyvinyl alcohol. In addition, it can be seen from the calculus curve DTG of TG, namely the curve obtained by the first derivative of each time coordinate on TG curve, that the weight change rate of GO–CE–CS–PVA at this stage is generally lower than that of GO–CS–PVA, which is owing to the modification of the carbon material GO. At the same time, after 800 °C high temperature treatment, the quality of GO–CE–CS–PVA still retains 22%. Therefore, compared with GO–CS–PVA, GO–CE–CS–PVA has relatively higher thermal stability.

### 3.2. Adsorption Experimental Analysis

#### 3.2.1. pH Experiment

**Figure 6:**
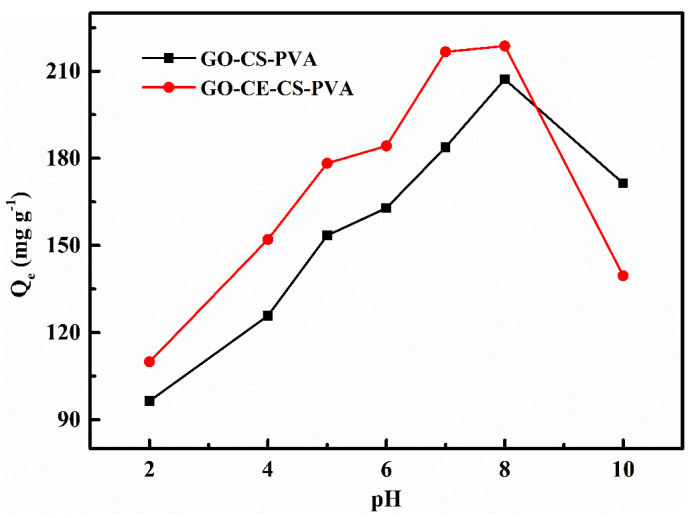
The effect of pH on the adsorption of Li^+^ by GO–CE–CS–PVA and GO–CS–PVA.

Because the surface properties of the adsorbent will be different under different pH conditions, the pH in the solution will have a great impact on the adsorption effect. In this work, under the condition that the initial concentration of Li^+^ is 1000 mg/L, the adsorption time is 24 h, and the adsorbent is 10 mg; we reacted two adsorbents, GO–CS–PVA and GO–CE–CS–PVA, in seven lithium ion solutions with different pH values. Taking into account the actual acidity and alkalinity of the salt lake brine, the pH range of this experiment is set to 2.0–10.0. It can be seen from Figure 6 that, with the increase in pH value, the overall trend of the adsorption capacity of GO–CS–PVA and GO–CE–CS–PVA on Li^+^ also increases. Under acidic conditions, there is a large amount of H^+^ in the solution, and it is easy to combine with the lone pair of electrons of –H_2_ through the coordination bond to form –NH^3+^. When the –NH_2_ of CS is protonated to –NH^3+^, the chelating ability with metal ions is reduced. In addition, the –O roots and –OH roots of graphene oxide are also easy to combine with H^+^ under acidic conditions, which will strengthen the reduction and reduce the effective adsorption sites on the surface of the graphene oxide. In addition, under alkaline conditions, when the pH value is greater than 8.0, the adsorption capacity of GO–CS–PVA and GO–CE–CS–PVA on Li^+^ drops significantly. This is because of the fact that lithium ions easily combine with –OH radicals to form lithium hydroxide inorganic compounds under alkaline conditions. Therefore, we choose pH = 7.0 as the best condition for subsequent experiments.

#### 3.2.2. Adsorption Kinetics Experiment

**Figure 7:**
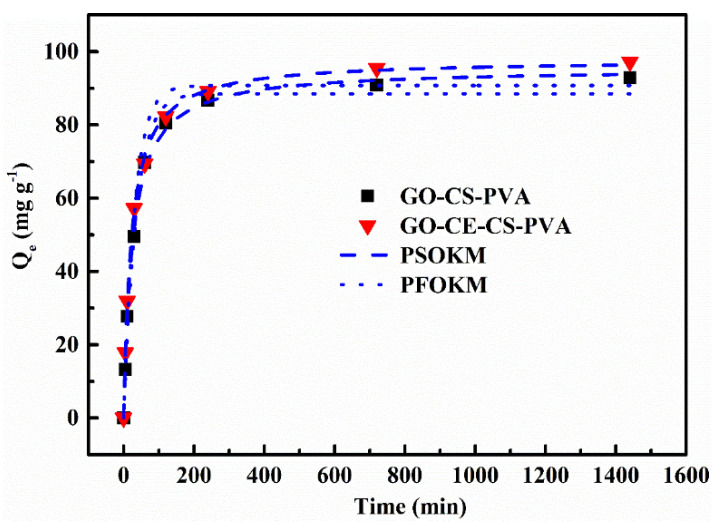
The adsorption kinetic data and model of Li^+^ by GO–CE–CS–PVA and GO–CS–PVA.

We examined the adsorption performance of GO–CE–CS–PVA for Li^+^ in different contact times to explore when the membrane can reach adsorption equilibrium during the adsorption process. We used the quasi-first-order kinetic model (PFOKM) and the quasi-second-order kinetic model (PSOKM) to perform nonlinear fitting to the experimental data of kinetic adsorption. We are under the conditions of 298 K, pH = 7.0, and 1000 mg L^−1^, and eight sets of experimental sampling points were set up within 0–140 min. After sampling, ICP was used to determine the concentration of remaining Li^+^ in the solution. The specific experimental results are shown in Figure 7.

From Figure 7, we can see that the adsorption capacity of GO–CE–CS–PVA on Li^+^ is generally higher than that of GO–CS–PVA. This shows that the unique selectivity of the added crown ether helps the synergistic adsorbent to capture the target metal ion Li^+^ quickly and efficiently. In addition, within 0–60 min, the adsorption capacity of either GO–CE–CS–PVA or GO–CS–PVA for Li^+^ increases rapidly. At the same time, the adsorption reached equilibrium in about 120 min. The related kinetic fitting parameters are shown in Table 1. It can be seen from the table that the correlation coefficients of the quasi-second-order kinetic model of the two materials are higher than those of the quasi-first-order kinetic model, which are 0.996 and 0.998, respectively. This shows that chemical adsorption is the mainstay in the whole adsorption process, and physical adsorption caused by electrostatic force is the auxiliary function.

#### 3.2.3. Adsorption Isotherm Experiment

**Figure 8:**
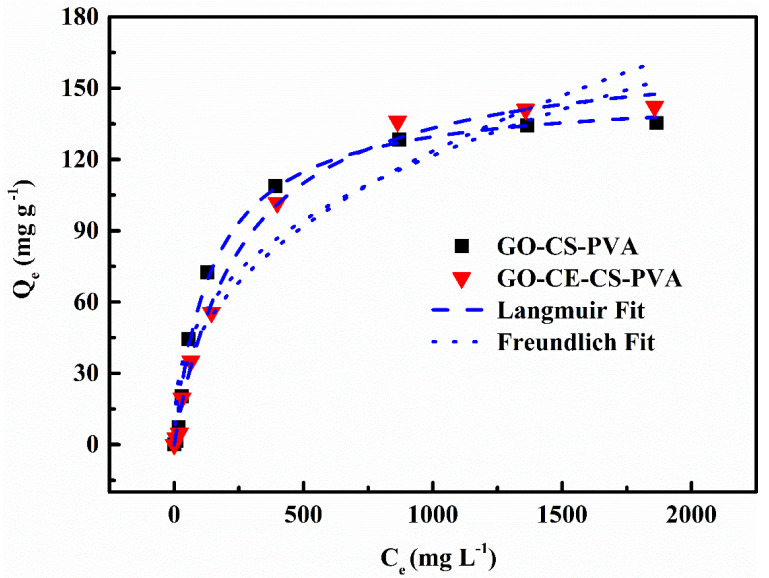
The adsorption isotherm data and model of GO–CE–CS–PVA and GO–CS–PVA for Li^+^.

In order to explore the maximum adsorption capacity of the prepared adsorbent for Li^+^, we carried out adsorption isotherm experiments in different concentrations of Li^+^ solution to obtain its maximum adsorption capacity. The Langmuir isotherm model and the Freundlich isotherm model were used to fit the experimental data. The specific fitting results are shown in Figure 8. It can be clearly seen from the figure that the fitting results of the adsorption isotherms of GO–CE–CS–PVA and GO–CS–PVA are more in line with the Langmuir isotherm model. The correlation coefficients are 0.994 and 0.992, respectively (see Table 2). This shows that the entire adsorption is a process dominated by single-layer chemical adsorption, all adsorption sites have the same probability, and the adsorbed lithium ions are completely independent. In addition, it can be seen from Table 2 that the maximum adsorption capacity of GO–CE–CS–PVA is 168.50 mg·g^−1^, which is higher than that of GO–CS–PVA, which confirms that crown ether can effectively help the adsorbent improve the adsorption capacity in the adsorption process.

#### 3.2.4. Adsorption Thermodynamics Experiment

**Figure 9:**
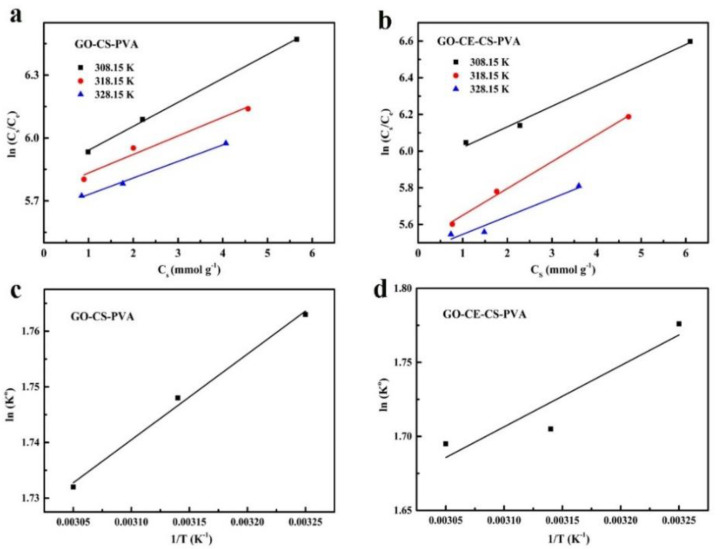
Plots of ln (Cs/Ce) as a function of Cs at different temperatures (308.15 K, 318.15 K, and 328.15 K): (**a**) GO–CS–PVA and (**b**) GO–CE–CS–PVA. ΔH^0^ and ΔS^0^ were obtained by linear fitting plots ln K^0^ against 1/T: (**c**) GO–CS–PVA and (**d**) GO–CE–CS–PVA.

In order to explore the thermodynamic properties of adsorption, we observed the change in adsorption efficiency of GO–CE–CS–PVA and GO–CS–PVA at different temperatures. Three temperatures of 308.15 K, 318.15 K, and 328.15 K were selected for thermodynamic experiments. The linear fitting of specific experimental data is shown in Figure 9. From the figure, we can see that, as the temperature increases, the adsorption capacity of the two materials decreases accordingly, which may be caused by the instability of CS at high temperatures. In addition, Table 3 shows that ΔH < 0 for both materials, indicating that the adsorption process is an exothermic reaction, and ΔG < 0, indicating that the adsorption reaction is spontaneous. The a and b diagrams in Figure 9 also confirm that the adsorption process of lithium ions by GO–CE–CS–PVA and GO–CS–PVA is a spontaneous exothermic reaction.

#### 3.2.5. Selective Adsorption Experiment

**Figure 10:**
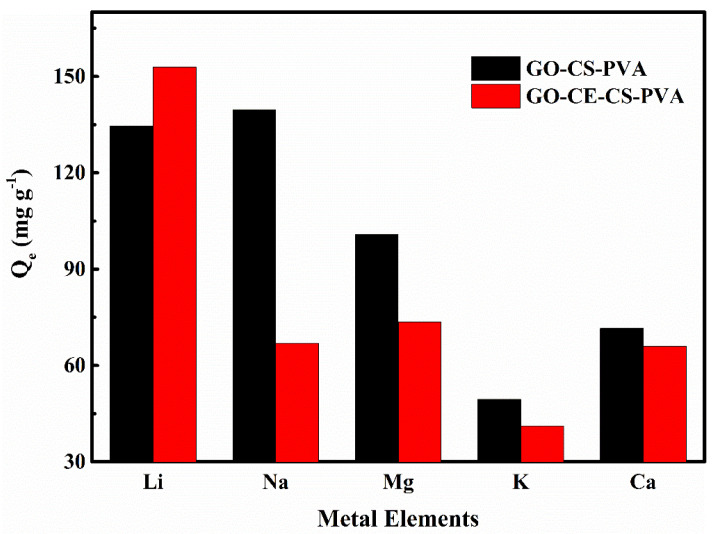
The selective adsorption diagram of GO–CE–CS–PVA and GO–CS–PVA for Li^+^ and other ions.

In China, rich salt lake brine resources have become the main direction of lithium extraction. However, the composition of salt lake brine is usually complex, and there are many interfering ions. Therefore, it is necessary to conduct selective adsorption experiments on adsorption materials. In this study, we selected four common interfering ions in salt lake brines—Na^+^, Mg^2+^, K^+^, and Ca^2+^—for adsorption selectivity experiments. It can be clearly seen from Figure 10 that the selectivity of GO–CE–CS–PVA nanofiber membrane to the target ion Li^+^ is significantly higher than that of GO–CS–PVA. The separation coefficients of Li^+^/Na^+^, Li^+^/Mg^2+^, Li^+^/K^+^, and Li^+^/Ca^2+^ are 2.51, 2.27, 4.19, and 2.55, respectively. It is about twice that of GO–CS–PVA (see Table 4). This is attributed to the GO–CE–CS–PVA surface grafted with a crown ether with a unique cavity structure, and the hole diameter of 2H12C4 (1.2–1.5 Å) is very close to the ion radius of Li^+^ (0.68 Å), which can be highly efficient to quickly capture Li^+^. Therefore, the above results indicate that GO–CE–CS–PVA has the strongest affinity for Li^+^ in the mixed solution with other interfering ions.

#### 3.2.6. Repeatable Adsorption Experiment

**Figure 11:**
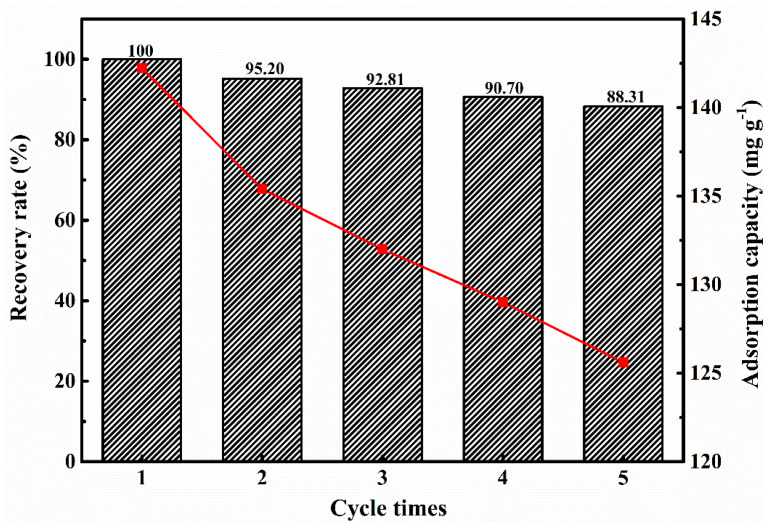
Repeated adsorption experiment of Li^+^ by GO–CE–CS–PVA.

In order to further verify the stability of the material in practical industrial applications, we conducted five repeated experiments on the adsorption performance of GO–CE–CS–PVA nanofiber membranes. The adsorption film was removed by acid pickling, adsorbed under the same conditions after elution. The cycle was repeated five times, and the adsorption amount was recorded each time. The specific results are shown in Figure 11. It can be seen from the figure that, after five cycles, the adsorption performance of the GO–CE–CS–PVA nanofiber membrane decreases slightly. It may be because of incomplete desorption of lithium ions or loss of adsorption sites during the elution process, which affects the cycle performance. However, after five cycles, the GO–CE–CS–PVA nanofiber membrane still maintains 88.31% of the adsorption capacity, which shows that the GO–CE–CS–PVA nanofiber membrane still has promising industrial application prospects.

## 4. Conclusions

In summary, we adopted a step-by-step method, combined with CE modified functionalized GO, CS, and PVA macromolecular materials, and the GO–CE–CS–PVA nanofiber membrane with excellent performance was successfully prepared by low temperature thermally induced liquid–liquid phase separation technology, which achieved high-efficiency and selective adsorption and separation of Li^+^. A series of characterizations proved the superior mechanical properties of the GO–CE–CS–PVA nanofiber membrane. The specific surface area of the GO–CE–CS–PVA nanofiber membrane is as high as 101.5 m^2^ g^−1^. A series of static and dynamic adsorption experiments and simulated adsorption results of actual interference ions show that the maximum adsorption capacity of the GO–CE–CS–PVA nanofiber membrane can reach 168.50 mg g^−1^ when the pH is 7.0. The adsorption kinetics and isotherm fitting results show that the adsorption mechanism of the GO–CE–CS–PVA nanofiber membrane to Li^+^ belongs to single-layer chemical adsorption. In addition, the excellent selective adsorption of Li^+^ and good reusability are affirmation of the stability of the GO–CE–CS–PVA nanofiber membrane, which also makes it more valuable in the application of lithium extraction from salt lake brine.

## Figures and Tables

**Table 1:** PFOKM and PSOKM parameters of GO–CE–CS–PVA and GO–CS–PVA.

Adsorbents	*Q*_e,exp_ (mg g^−1^)	PFOKM			PSOKM				
		*Q*_e,c_ (mg g^−1^)	*k*_1_ (min^−1^)	*R* ^2^	*Q*_e,c_ (mg g^−1^)	*K*_2_ × 10^−2^ (g mg^−1^ min^−1^)	*h* (mg g^−1^ min^−1^)	*t*_1/2_ (min)	*R* ^2^
GO-CS-PVA	92.82	88.49	0.028	0.983	95.42	0.041	3.77	25.28	0.996
GO-CE-CS-PVA	97.23	90.76	0.032	0.954	97.83	0.046	4.43	22.07	0.998

**Table 2:** Langmuir and Freundlich parameters of GO–CE–CS–PVA and GO–CS–PVA.

Adsorbents	Langmuir Isotherm Equation				Freundlich Isotherm Equation		
	*R* ^2^	*K*_L_ (L mg^−1^)	*Q*_m_ (mg g^−1^)	*R*_L_	*R* ^2^	*K*_F_ (mg g^−1^)	1/*n*
GO-CS-PVA	0.992	0.007	148.75	0.069	0.900	9.69	0.366
GO-CE-CS-PVA	0.994	0.004	168.50	0.117	0.933	6.46	0.427

**Table 3:** The adsorption thermodynamic parameters of GO–CE–CS–PVA and GO–CS–PVA for Li^+^.

Adsorbents	∆*H* (kJ mol^−1^)	∆*S* (J mol^−1^)	*T* (K)	*K* ^o^	∆*G* (kJ mol^−1^)
GO-CS-PVA	−1.28	10.49	308.15	5.83	−4.516
			318.15	5.74	−4.624
			328.15	5.65	−4.725
GO-CE-CS-PVA	−3.44	3.52	308.15	5.91	−4.551
			318.15	5.50	−4.511
			328.15	5.45	−4.625

**Table 4:** The selective adsorption parameters of GO–CE–CS–PVA and GO–CS–PVA for Li^+^ and other ions.

Metal Ions	GO-CS-PVA			GO-CE-CS-PVA		
	*C*_f_ (mg·L^−1^)	*K*_d_ (mL·g^−1^)	*k*	*C*_f_ (mg·L^−1^)	*K*_d_ (mL·g^−1^)	*k*
Li^+^	865.38	0.156	/	847.13	0.180	/
Na^+^	860.35	0.162	0.96	933.08	0.072	2.51
Mg^2+^	899.08	0.112	1.39	926.49	0.079	2.27
K^+^	950.56	0.052	3.00	958.81	0.043	4.19
Ca^2+^	928.48	0.077	2.03	933.96	0.071	2.55
